# How academic stress leads to artificial intelligence-generated design dependency: the roles of academic procrastination and help-seeking behavior

**DOI:** 10.3389/fpsyg.2026.1794730

**Published:** 2026-03-18

**Authors:** Dan Wang, Zhenliang Wang

**Affiliations:** 1 Art Design College, Henan University of Urban Construction, Pingdingshan, China; 2 School of Ceramic, Pingdingshan University, Pingdingshan, China

**Keywords:** academic help-seeking behavior, academic procrastination, academic stress, AIGD dependency, AI-generated design

## Abstract

Against the background that design students generally rely on artificial intelligence-generated design (AIGD) to address academic challenges, concerns have arisen about the potential dependency risks posed by its improper use. Therefore, based on the Interaction of the Person-Affect-Cognition-Execution (I-PACE) model, this study explores the association between academic stress and AIGD dependency among design students and investigates the chain mediation effects of academic procrastination and academic help-seeking behaviors. To solve the problem that academic stress may cause students’ AIGD dependency, this study clarified the mechanism from procrastination to seeking academic help caused by academic stress and suggested feasible intervention goals to prevent the use of AIGD from evolving into dependency. The data analysis of 492 design university students indicates that academic stress is positively associated with AIGD dependency. Academic procrastination and academic help-seeking behaviors exert a significant effect on chain mediation. The research results explain the psychological mechanism by which AIGD evolves from an auxiliary tool into a dependency object under high academic stress. This study extends the application of the I-PACE framework to AIGD dependency in design education. The results offer a theoretical explanation for understanding the psychological mechanism of AIGD dependency among design students and provide practical implications for educational interventions.

## Highlights

- This study applies the I-PACE framework to examine how academic stress is associated with AIGD dependency among design students.
- Academic procrastination and help-seeking behavior serially mediate the relationship between academic stress and AIGD dependency.
- A serial mediation path shows that academic stress is linked to higher procrastination, which is related to maladaptive help-seeking and is further associated with AIGD dependency.
- Findings expand the I-PACE framework to AIGD dependency and highlight how stress-related behaviors related to students’ reliance on AI tools.

## 1 Introduction

As the core driving force of contemporary society, the rapid development of artificial intelligence (AI) technology is reshaping global socioeconomic structures and individual lifestyles while bringing both opportunities and challenges (Lund and Wang, 2023). AIGD represents a model of human-computer collaboration. In the design process, tools such as Midjourney, DALL-E, Stable Diffusion, and Canva serve as “collaborators,” leveraging perception, learning, reasoning, and image generation to enhance designers’ and learners’ capabilities (Peckham et al., 2025). Based on deep learning models, such as potential diffusion, these systems convert text prompts into professional-grade visual patterns, thereby enabling image-creation automation (Zhou and Lee, 2024). As an effective assistance tool, the reasonable use of AIGD offers students a convenient design support system. However, many students tend to use these tools as a shortcut to escape deep thinking, thus leading to dependency (Doshi and Hauser, 2024). This dependence regards AIGD as an efficient external tool for research and specific tasks. When facing academic difficulties, AIGD could help students manage or even complete complex design assignments (Huang et al., 2024). The survey shows that students use AIGD to reduce the time spent on independent conception. This reliance intensifies under time pressure, particularly near the submission time (Saadi and Yang, 2023; Yu et al., 2025). Using AIGD to optimize designs may effectively improve assignment completion and boost short-term academic performance. This positive feedback will strengthen students’ usage behavior, making them more likely to rely on AIGD and avoid independent deep thinking (Zhang and Zhang, 2025). Various phenomena show that design students have become dependent on AIGD (Xu, 2025).

In this study, “AIGD dependency” refers to a pattern of excessive reliance on AIGD tools in assisting or completing academic design tasks, reflected in discomfort when the tools are unavailable and a belief that task quality cannot be guaranteed without these tools. This concept is distinct from general AIGD use, described as a measurable dependence tendency such as problematic overreliance in the educational context rather than clinical addiction (Goh et al., 2025; Morales-García et al., 2024).

Previous research indicates that university students generally experience academic stress, which affects their academic performance (Dalvi-Esfahani et al., 2019; Guo et al., 2011; Jun and Choi, 2015; You, 2018). Academic stress may be a significant driver of students’ reliance on AIGD. The heavier the academic stress, the more obvious the impact. Common sources of stress include academic competition, performance expectations, career uncertainty, and economic burden (Lee et al., 2005). To alleviate academic stress, students resort to AIGD tools to improve their learning efficiency. Without educational intervention, a vicious circle of AIGD dependency may develop (Brosens et al., 2023). They seek quick solutions when facing academic difficulties, exacerbating AIGD dependency (Ungerer and Slade, 2022). Previous research confirms that the convenience of AI generative tools may aggravate overreliance (Frenkenberg and Hochman, 2025; Yankouskaya et al., 2025). Considering the potential dependence of design students on AIGD tools, guiding them to establish a healthy and sustainable collaborative relationship with AIGD is a crucial concern for design educators (Brosens et al., 2023). In general, there are some limitations in existing research. First, research on dependency in design education is scarce, even though the detrimental effects of excessive reliance on AIGD have garnered attention (Chaturvedi et al., 2024). Second, the existing literature has insufficiently examined how academic stress leads to AIGD dependency. To address research gaps, this study will explore the correlation between academic stress and AIGD dependency.

Based on this concern, this study aimed to clarify the mechanism between academic stress and AIGD dependency and offered feasible suggestions about preventing the use of AIGD from evolving into dependency. The Person-Affect-Cognition-Execution (I-PACE) model aims to explain personal problematic behaviors related to the Internet and technology applications (Brand et al., 2016). Grounded in this framework, the present study investigates the students’ AIGD dependency in the design educational context to explore how academic stress leads to AIGD dependency and test the mediating roles of academic procrastination and academic help-seeking behavior. This study provides empirical evidence for the rational use of AIGD in design education and expands the application scope of the I-PACE framework. Therefore, this study addresses the following research questions:

*RQ1:* Are students’ academic stress associated with AIGD dependency?

*RQ2:* Do academic procrastination and help-seeking behavior mediate the relationship between academic stress and AIGD dependency, and if so, how?

## 2 Literature review

### 2.1 The I-PACE model

The I-PACE model (Brand et al., 2019) is a theoretical framework to clarify Internet addiction behavior, which includes four elements: personal attributes (P), affects (A), cognition (C), and execution (E). The element P covers personal characteristics, such as personality and psychopathological characteristics, social cognition, and cognitive vulnerability. The element A includes the emotional factors that affect individual behavior, including emotional reactions and coping strategies to various stimuli. The element C comprises the cognitive processes and biases that influence individuals and shape experiences. Component E is about an individual’s executive behavior, including the extent of technology use, self-control capacity, and decision-making competence (Zhang et al., 2024). The complex interaction between the four elements could explain the formation mechanism of addictive behavior.

The I-PACE model offers an insightful theoretical foundation for understanding problematic technology use in multiple domains (Elhai et al., 2018; Zhang and Xu, 2025). Preliminary research has applied the model to investigate the relevant problems of AI dependency (Hu et al., 2023; Zhang and Zhang, 2025) and verified the applicability of the I-PACE model to the investigation of problematic AI use. This study attempts to use this model innovatively as a theoretical framework for examining AIGD dependency.

The I-PACE model consists of four key components; this study specifically focuses on the P, A, and E components. We tested a partial pathway that is most relevant to the research context. In the present study, the personal attribute (P) is a proximate triggering factor, which confirmed to be an important trigger for behavioral problems (Zhang and Zhang, 2025). The dependent variable (E) represents executive outcomes, which specify AIGD dependency. While procrastination and help-seeking behaviors reflect cognitive coping responses (A), which may develop into AIGD dependency. In this study, academic procrastination and academic help-seeking behavior are identified as the key mediating variables. Specifically, academic procrastination intensifies students’ anxiety (emotion) and self-denial (cognition), making reliance on AIGD an evasive coping strategy. This framework helps explain why AIGD dependency may not only be generated through academic stress, but also operate through a series of behavioral mechanisms. Therefore, this study partially adopts the I-PACE model, which is consistent with the practice of testing key mechanisms in existing empirical studies (Hu et al., 2023; Marengo et al., 2020; Wegmann et al., 2017; Zhang and Zhang, 2025; Ye et al., 2025). Therefore, based on the I-PACE model, this study examines the specific path of P → A → E in a specific context.

### 2.2 Academic stress and AIGD dependency

Academic stress is the psychological burden experienced by students while striving to achieve academic objectives, characterized by the perceived disparity between the demands of academic tasks and their self-assessed capabilities, frequently accompanied by distinct cognitive, emotional, and physiological responses (McGrath, 1976; Struthers et al., 2000). It may contribute to psychological and behavioral problems among students (Jayasankara Reddy et al., 2018). This study identifies academic stress as a social factor leading to the use of problematic technology. Early studies demonstrate a positive correlation between students’ academic stress and academic procrastination, indicating that students experiencing high levels of stress show increased procrastination (Niazov et al., 2022; Rahardjo et al., 2013).

Stress-coping theory (Wills et al., 2001) points out that individuals tend to employ various strategies when facing stressful situations. On the contrary, ineffective coping mechanisms may foster problematic or addictive behavior (Metzger et al., 2017). In this study, AIGD not only acts as a tool to improve efficiency, but is also regarded as a learning assistance resource that may generate dependence under increased academic stress. Students experiencing higher academic stress demonstrate greater reliance on AIGD as a coping strategy, which may develop into excessive dependency (Rahman and Watanobe, 2023). The relevant study on AI-assisted learning suggests that higher academic stress is associated with stronger dependence on AI tools (Acosta-Enriquez et al., 2025; Liu et al., 2026). Therefore, we propose:

*H1*: Academic stress is positively associated with AIGD dependency.

### 2.3 Mediating role of academic procrastination

Procrastination constitutes a prevalent behavior pattern among university students. It describes the behavior of individuals who recognize that procrastination leads to negative outcomes but still choose to delay tasks until they experience subjective discomfort (Solomon and Rothblum, 1984). Research shows that academic procrastination negatively impacts academic performance and even leads to dropouts. Students’ procrastination tends to intensify as they progress through successive academic stages, with seniors displaying the highest degrees of such behavior (Semb et al., 1979). The I-PACE framework confirms a strong correlation between technology-related issues and procrastination (Brand et al., 2016). Previous studies have demonstrated that academic stress intensifies academic procrastination (Grunschel et al., 2013; Rahardjo et al., 2013). In addition to procrastination and other negative coping strategies, students increasingly adopt AIGD tools to actively seek academic help, which has become an alternative way to solve problems.

Previous research indicated that GenAI tools can improve students’ academic performance and engagement (Polak et al., 2022). These tools’ accessibility facilitates inexperienced educators and students in quickly mastering relevant skills and substantially enhancing educational outcomes (Polak et al., 2022). AIGD tools provide instant feedback and intelligent support to satisfy students’ diverse task demands (Wecks et al., 2024). Meanwhile, such convenience entails risks. Confronted with time constraints and task-specific anxiety caused by procrastination, the efficiency and convenience of the AIGD tool may emerge as the preferred recourse for individuals to seek solutions and alleviate stress. Considering that students experiencing greater academic stress exhibit significant academic procrastination. They may develop stronger reliance on AIGD as a coping mechanism. Therefore, we put forward the following hypothesis:

*H2*: Academic procrastination mediates the association between academic stress and AIGD dependency.

### 2.4 Mediating role of academic help-seeking behavior

During the learning process, students often confront complex learning tasks. Research indicates that most university students require additional support in specific courses or learning methods each semester (Karabenick and Knapp, 1988). However, even when aware that their learning difficulties are solvable, most students are unwilling to seek help (Nelson-Le Gall, 1987). Research has confirmed the adverse effects of avoiding seeking academic help. Academic help-seeking behavior can be divided into two categories: executive help-seeking, which directly obtains answers, and instrumental help-seeking, which aims to understand processes (Nadler et al., 2010). The instrumental approach represents the more adaptive strategy. In actual classroom settings, students voluntarily seek help relatively infrequently, with most relevant help-seeking taking place beyond formal instruction (Gall and Glor-Scheib, 1985). Within the I-PACE model framework (Brand et al., 2019), academic help-seeking functions as observable behavior motivated by affective-cognitive processes (A). Students with positive learning attitudes are more inclined to seek academic help when facing challenges; such support facilitates the attainment of academic goals (McKeachie et al., 1985). AIGD offers procedural guidance and immediate feedback, creating an effective channel for instrumental help-seeking among design students (Yang, 2025). By using the AIGD tool, students can get more in-depth insights and solutions, making it a valuable resource for both academic and psychological support. However, procrastination caused by academic stress exacerbates academic difficulties and anxiety, leading to an increasing reliance on AIGD as an external support that may potentially cause dependency. Therefore, we propose the following hypothesis:

*H3*: Academic help-seeking behavior mediates the association between academic stress and AIGD dependency.

### 2.5 Serial mediating roles of academic procrastination and academic help-seeking behavior

Academic procrastination frequently co-occurs with negative emotional states, including anxiety during decision-making, as well as feelings of guilt and discomfort after task completion (Shin and Jeon, 2011). When design students experience high levels of academic stress, they often exhibit pronounced procrastinatory tendencies that trap them in a passive situation. They subsequently seek assistance to break this cycle (Yang, 2025). In response, they express a preference for adopting AIGD as the primary academic support resource. This dependence constantly strengthens usage habits, and when confronted with similar academic challenges, such usage patterns solidify into psychological dependency. Against this background, this study believes that academic stress promotes academic help-seeking behavior by aggravating academic procrastination, which eventually leads to AIGD dependency. Therefore, we put forward the following hypothesis:

*H4*: Academic procrastination and academic help-seeking behaviors serially mediate the relationship between academic stress and AIGD dependency.

## 3 Methods

### 3.1 Sample and data collection

In September 2025, after approval from our university’s Institutional Review Board, we conducted an online survey to collect data from students in design majors at Chinese universities. This survey did not address sensitive or offensive topics. To improve accessibility for participant engagement. We used the “Wenjuanxing”^1^ questionnaire collection platform for data collection; it is a professional Chinese survey platform.

The survey adopts a non-probability purposive sampling strategy. We invited university students from various regions in China to participate in this survey, with the assistance of design university teachers. The participants are design undergraduates from public universities from 16 provinces across the country. The questionnaire links were distributed via WeChat. Participation was voluntary and anonymous, and each participant received a small incentive. Before data collection, we explained AIGD tools to participants and raised a screening question: “Have you used AIGD tools to complete design tasks in the past six months?” Only students reporting prior use could proceed.

The survey lasted for 2 weeks and yielded 570 online questionnaires. During data cleaning, we exclude the same answers and invalid questionnaires among all scale items. The final sample includes 492 valid questionnaires for subsequent statistical analysis. Table 1 presents the sample’s demographic characteristics.

**Table 1:** Sample characteristics and frequency analysis results (*N* = 492).

		Number	Percentage
Gender	Female	235	47.76
	Male	257	52.24
Grade	Freshman	105	21.34
	Sophomore	130	26.42
	Junior	135	27.44
	Senior	122	24.8
Types of Universities	Comprehensive university	132	26.83
	University of Science and Technology	116	23.58
	Normal university	115	23.37
	Other universities	129	26.22
Major	Visual Communication Design	72	14.63
	Product design	99	20.12
	Animation	90	18.29
	Environmental design	69	14.02
	New media design	78	15.85
	Fashion design	84	17.07
Total		492	100

Main reasons for using AIGD tools	
Time-saving and efficiency improvement	89.84%
Inspiration acquisition and overcoming creative hurdles	75.61%
Accomplish complex design tasks,	72.56%
The prevailing trend of AIGD adoption	40.45%
Curiosity about emerging technologies	36.18%
AIGD Tools’ impact on capabilities	
Enhancement of creative thinking capabilities	29.88%
Development of hands-on skills	28.46%
Advancement of software operation proficiency	24.59%
Strength of critical thinking	27.03%
Promotion of self-learning capacity	26.63%

### 3.2 Instruments development

In this study, the questionnaire consists of two parts. The first part aims to collect participants’ basic demographic information, including their gender, major, age, and experience using AIGD. The second part contains four component scales, with a total of 16 items. These items were adapted from validated instruments to measure the study’s key variables. The questionnaire was translated into Chinese by a professional translator. We invited three design professors to review each item and confirm the clarity and suitability for the respondents’ learning context. Before the survey, we conducted a preliminary test with 30 students to assess the comprehensibility and feasibility. We made minor revisions according to their feedback. Then, the reliability and validity of the main samples were tested. All measures used a seven-point Likert scale ranging from 1 (strongly disagree) to 7 (strongly agree).

### 3.3 AIGD dependency (Cronbach’s *α* = 0.842)

The AIGD dependency scale was adapted from Morales-García et al. (2024) measure of AI dependency (Cronbach’s α = 0.87). Although the original scale was developed in Spanish, its validity has been established in a study of inertia thinking and ChatGPT dependency with Chinese university students. For our purposes, the term “artificial intelligence” was replaced with “AIGD,” resulting in four items. Sample items include “I feel uneasy when unable to use AIGD tools,” “I worry my designs or tasks won’t meet expectations without AIGD”, “I continuously follow the latest developments in AIGD to ensure that I remain aligned with current trends in the design field,” and “I feel more confident in my decisions only after receiving feedback from AIGD.” In this study, the scale demonstrated adequate reliability (Cronbach’s *α* = 0.842).

#### 3.3.1 Academic stress (Cronbach’s α = 0.864)

The academic stress scale was adapted from Jun and Choi's (2015) work on academic stress and internet addiction (Cronbach’s α = 0.81). This scale was adapted from the research of Park et al. (2008). This measurement was adopted in a recent South Korean study investigating the association between social capital and academic stress (Jarvis et al., 2020). This adaptation scale consists of four items, such as “I am deeply concerned about my academic performance”, “Heavy design assignment makes me feel stressed,” “My desire to complete my studies successfully makes me feel stressed,” and “I feel exhausted with my studies.” The scale showed strong reliability in the present research (Cronbach’s *α* = 0.864).

#### 3.3.2 Academic procrastination (Cronbach’s *α* = 0.859)

The four-item measure of the Academic Procrastination Scale, adapted from Solomon and Rothblum’s (1984) Student Procrastination Assessment Scale, has a Cronbach’s α of 0.88. This scale has been widely validated in previous research. In the present study, the adapted scale included “I tend to procrastinate when working on design projects,” “I often postpone preparation for examination,” “I tend to procrastinate when completing course assignments,” and “I tend to procrastinate when writing academic papers.” The scale showed a strong reliability (Cronbach’s α = 0.859).

#### 3.3.3 Academic help-seeking behavior (Cronbach’s α = 0.850)

The four-item measure of the Academic Help-Seeking Behavior Scale was adapted from Karabenick and Knapp’s (1988) study; it has been widely applied and was previously validated in a study examining college students’ academic help-seeking and learning strategies (Karabenick and Knapp, 1991). In this study, the adapted scale included four items, including “At least one course I am taking this semester necessitates additional academic support,” “In the face of academic challenges, I tend to seek the help of teachers.” “When I encounter difficulties in my learning, I tend to seek help from peers,” and “When I encounter difficulties in my learning, I tend to seek help from Internet tools. The scale demonstrated adequate reliability (Cronbach’s α = 0.850).

## 4 Data analysis

**Figure 1:**
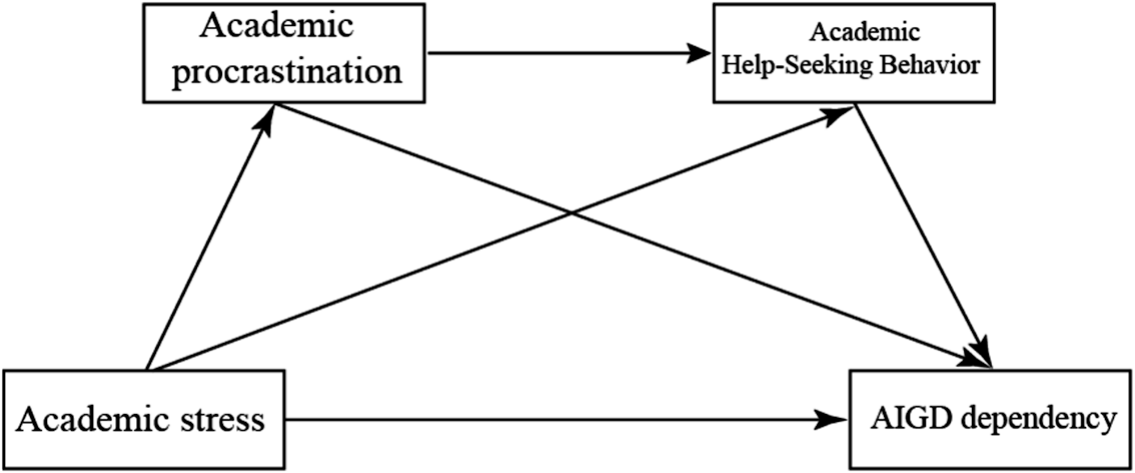
The research model.

The data analysis was conducted using SPSS 25.0 and AMOS 26.0. Structural equation modeling (SEM) and confirmatory factor analysis (CFA) are used because all key variables are latent variables measured by multiple indicators, CFA evaluates the measurement model, and SEM can estimate structural relationships. During the analysis, we tested the hypothesized model as shown in Figure 1. The analytical procedure unfolded in several stages. First, CFA was conducted to evaluate the reliability and validity of the measurement instrument. Cronbach’s alpha coefficients were calculated to test the internal consistency of each subscale; these steps were used to validate the measurement model. Next, SEM was used to verify the hypothesized structural model. The results are presented in Table 2. Finally, the PROCESS macro (Model 6) was used to test the proposed mediation pathway, including the mediating roles of academic procrastination and academic help-seeking behavior in the relationship between academic stress and AIGD dependency. The indirect effect was tested through 5,000 bias-corrected bootstrap samples with corresponding 95% confidence intervals (CIs). Indirect effects were considered significant when the 95% bootstrap confidence interval for the indirect effect did not include zero.

**Table 2:** Fit indices for the measurement and structural models.

Model	χ ^2^	χ ^2/df^	AGFI	NFI	TLI	CFI	RMSEA
Measurement model	261.336 (*p* < 0.001)	2.667	0.920	0.937	0.950	0.959	0.058
Structural model	261.336 (*p* < 0.001)	2.667	0.920	0.937	0.950	0.959	0.058
Recommended criteria	*/*	<3.0	>0.90	>0.90	>0.95	>0.95	<0.08

*χ*^2^, chi-square; df, degrees of freedom; AGFI, adjusted goodness-of-fit index; NFI, normed fit index; TLI, Tucker–Lewis index; CFI, comparative fit index; RMSEA, root mean square error of approximation.

Before conducting hypothesis testing, we screened the collected data and checked for missing values and abnormal response patterns. We conducted Harman’s single-factor test to examine the potential common method bias. The unrotated exploratory factor solution showed that the first factor accounts for 36.9% of the total variance, which was below the 40% threshold, indicating that common method bias was unlikely to seriously threaten the interpretation of the findings.

## 5 Results

### 5.1 Frequency results

In terms of sample demographics, male respondents outnumbered females. Respondents aged 21 accounted for the largest proportion (21.7%), while third-year university students made up the highest proportion (27.44%). Regarding the institution types, the number of students in comprehensive universities represented the majority at 26.83%. Regarding the participants’ academic major composition, product design accounted for the largest proportion (20.1%). Overall, the sample is broadly distributed regarding grade, university type, and design major, providing diversity for subsequent analysis.

The analysis of the survey data identified the main reasons and motivations for design students using AIGD tools. The functions that respondents use the most frequently are text generation (80%), design assistant (76.2%), image generation (72.6%), and video generation and editing (69.1%). AIGD is mostly used in academic assignments for copywriting (76%), inspiration and brainstorming (74.1%), and portfolio creation (70.53%), data collection and preliminary research (68.2%). Regarding usage motivations, the main reasons were to improve efficiency and save time (89.8%), to gain inspiration and creativity (75.6%), and to complete difficult assignments (72.6%). while 40.5% of the respondents indicated being influenced by peer behavior, 36.2% reported curiosity about emerging technologies. The survey data show that AIGD tools are primarily used to improve the efficiency of academic tasks, with a secondary motivation being to seek support for creativity.

In addition, participants reported positive developments in self-perceived competence using AIGD. Specifically, 29.9% of respondents reported an improvement in creative thinking, 28.5% reported an enhancement in hands-on skills, 27% noted a strengthening of critical thinking, and 26.6% reported an increase in self-learning.

### 5.2 Descriptive statistics, correlations, and measurement model evaluation

There is a positive correlation between academic stress (mean = 4.19, SD = 1.38), academic procrastination (mean = 4.20, SD = 1.37), academic help-seeking behavior (mean = 4.12, SD = 1.34), and AIGD dependency (mean = 4.17, SD = 1.26). All variables showed positive correlations. Specifically, academic stress demonstrated significant positive correlations with academic procrastination (*r* = 0.310, *p* < 0.05) and academic help-seeking behavior (*r* = 0.326, *p* < 0.05), and AIGD dependency (*r* = 0.368, *p* < 0.05). Both academic procrastination (*r* = 0.392, *p* < 0.05) and academic help-seeking behavior (*r* = 0.481, *p* < 0.05) were positively correlated with AIGD dependency. A significant correlation was observed between academic procrastination and academic help-seeking behavior (*r* = 0.317, *p* < 0.05), which is consistent with the proposed mechanism that students may use AIGD-generated content as an accessible academic support resource.

As shown in Table 3, the measurement model demonstrated acceptable reliability and convergent validity. Cronbach’s *α* ranged from 0.842 to 0.864, and Composite Reliability (CR) values ranged from 0.852 to 0.86, all above the recommended threshold of 0.70. Average Variance Extracted (AVE) metrics ranged from 0.593 to 0.624, all exceeding the recommended threshold of 0.50. standardized factor loadings ranging from 0.692 to 0.912.

**Table 3:** Reliability and convergent validity of the measurement model.

Latent variable	Measurement variable	Mean	Std. Dev	Factor loadings	α	CR	AVE
AS	AS1	4.19	1.38	0.885	0.864	0.868	0.624
	AS2			0.768			
	AS3			0.769			
	AS4			0.728			
AP	AP1	4.20	1.37	0.903	0.859	0.866	0.620
	AP2			0.743			
	AP3			0.739			
	AP4			0.753			
AHSB	AHSB1	4.12	1.34	0.898	0.850	0.857	0.603
	AHSB2			0.73			
	AHSB3			0.726			
	AHSB4			0.738			
AIGDD	AIGDD1	4.17	1.26	0.912	0.842	0.852	0.593
	AIGDD2			0.692			
	AIGDD3			0.728			
	AIGDD4			0.727			

AS, academic stress; AP, academic procrastination; AHSB, academic help-seeking behavior; AIGDD, AIGD dependency; M, mean value; SD, standard deviation; α, Cronbach’s Alpha; CR, composite reliability; AVE, average variance extracted.

As presented in Table 4, the model demonstrates adequate discriminant validity among the four constructs, satisfying the Fornell-Larcker criterion. In addition, the CFA measurement model showed acceptable fit (*χ^2^/df* = 2.667, *CFI* = 0.959, *TLI* = 0.950, *RMSEA* = 0.058); the fit indices are reported in Table 2. The results from Tables 2–4 indicate that the measurement model is appropriate and provides a reliable basis for testing the structural relationships of the hypothesis model.

**Table 4:** Discriminant validity (Fornell–Larcker criterion).

Constructs	AS	AP	AHSB	AIGDD
AS	**0.790**			
AP	0.310	**0.788**		
AHSB	0.326	0.317	**0.777**	
AIGDD	0.368	0.392	0.481	0.**770**

The bold values in the diagonal row are the square roots of the average variance extracted for the constructs in the research model.

### 5.3 Mediation analysis

The results of the chain mediation analysis for academic procrastination and AIGD dependency are presented in Table 5. All analyses were interpreted following the statistical guidelines proposed by Lee et al. (2021).

**Table 5:** Results of chain mediation analysis.

Dependent variable	Independent variable	*β*	*S.E*	*t*	95%CI	
					LLCI	ULCI
AP	AS	0.3075	0.0426	7.2181***	0.2238	0.3912
AHSB	AS	0.2443	0.0422	5.7852***	0.1613	0.3272
	AP	0.2325	0.0426	5.4610***	0.1488	0.3161
AIGDD	AS	0.1686	0.0370	4.5548***	0.0959	0.2414
	AP	0.2061	0.0372	5.5399***	0.1330	0.2792
	AHSB	0.3315	0.0384	8.6402***	0.2561	0.4068

AS, academic stress; AP, academic procrastination; AHSB, academic help-seeking behavior; AIGDD, AIGD dependency. ***p < 0.001, **p < 0.01, *p < 0.05.

The results revealed several key findings. (1) Academic stress had a significant positive direct effect on AIGD dependency (*β* = 0.1686, SE = 0.0370, 95% CI [0.0959, 0.2414]), thus supporting H1. (2) Academic stress was positively associated with academic procrastination (*β* = 0.3075, SE = 0.0426, 95% CI [0.2238, 0.3912]), and academic procrastination was significantly positively associated with AIGD dependency (*β* = 0.206, SE = 0.0372, 95% CI [0.1330, 0.2792]). The indirect effect of academic procrastination in the relationship between academic stress and AIGD dependency was significant (effect = 0.0634, 95% CI [0.0393, 0.0916]), thus supporting H2. (3) Academic stress was significantly positively correlated with academic help-seeking behavior (*β* = 0.2443, SE = 0.0422, 95% CI [0.1613, 0.3272]), and academic help-seeking behavior was a significant positive predictor of AIGD dependency (*β* = 0.3315, SE = 0.0384, 95% CI [0.2561, 0.4068]). The indirect effect of academic help-seeking behavior in the association between academic stress and AIGD dependency was significant (effect = 0.0810, 95% CI [0.0491, 0.1188]), supporting H3. (4) Academic procrastination was positively associated with academic help-seeking behavior (*β* = 0.2325, SE = 0.0426, 95% CI [0.1488, 0.3161]), the chain mediation effect of academic procrastination and academic help-seeking behavior in the relationship between academic stress and AIGD dependency was significant (effect = 0.0237, 95% CI [0.0137, 0.0362]), which supports H4.

**Figure 2:**
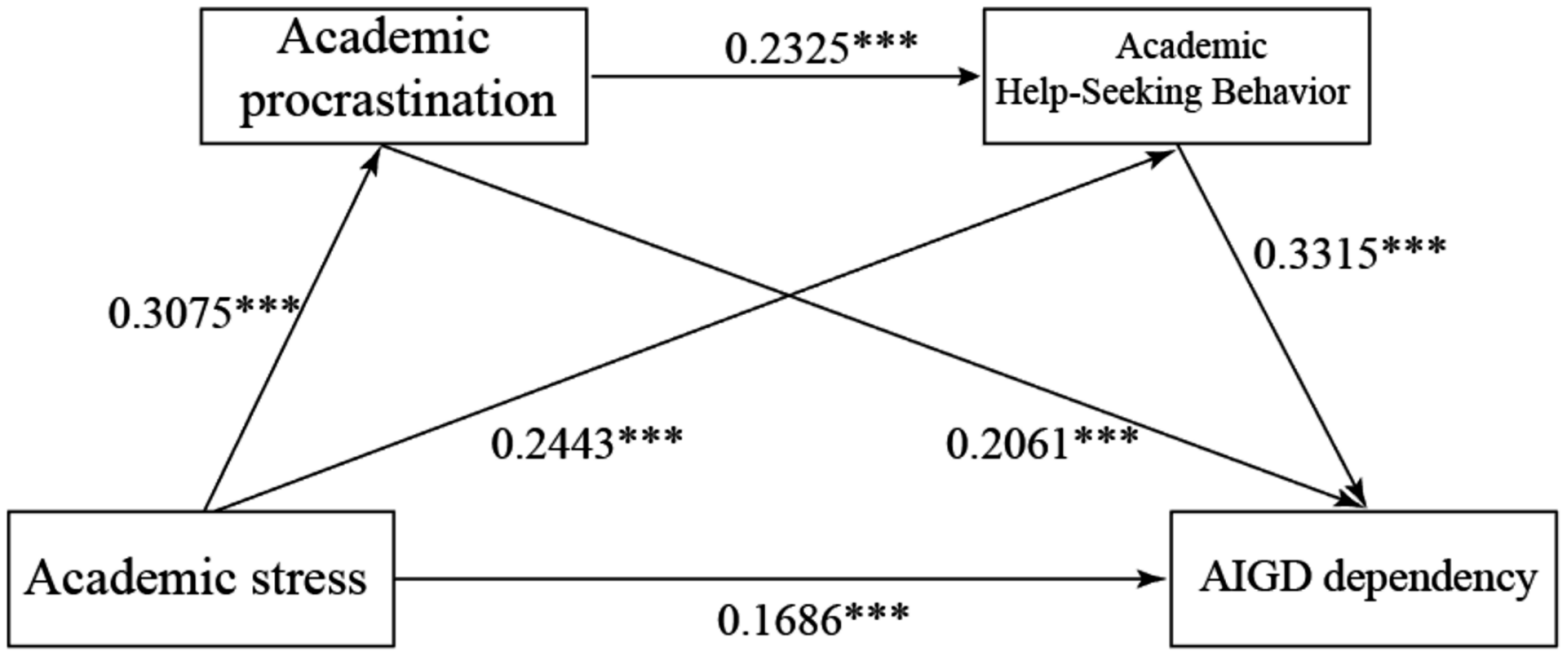
The research model with its standardized coefficients. ***p < 0.001, **p < 0.01, *p < 0.05.

Table 6 further summarizes the overall and indirect effects of academic stress on AIGD dependency, including the relative contributions of three indirect pathways. The total effect of academic stress on AIGD dependency was 0.3367, with the direct effect (0.1686) accounting for 50.1%, and the total indirect effect (0.1681) accounting for 49.9%. Among all indirect pathways, the indirect effect via academic help-seeking behavior was the largest at 48.2%, followed by academic procrastination at 37.7%, and the chain-mediated pathway had the smallest impact at 14.1%. The structural model explains 34.2% of the variance in AIGD dependency (*R*^2^ = 0.342), 9.2% of the variance in academic procrastination (*R*^2^ = 0.092), and 15.6% of the variance in academic help-seeking behavior (*R*^2^ = 0.156). In summary, these results support the hypothesis mechanism based on I-PACE, showing that academic stress is associated with AIGD dependency via two mediating roles. Figure 2 presents the standardized path coefficients for the hypothesized model.

**Table 6:** Direct and indirect effects of academic stress on AIGD dependency.

	Effect	SE	LLCI	ULCI	Proportion of total effect
Total effect of AS on AIGDD	0.3367	0.0384	0.2612	0.4122	
Direct effect of AS on AIGDD	0.1686	0.0370	0.0959	0.2414	50.1%
Total indirect effect	0.1681	0.0237	0.1240	0.2165	49.9%
Indirect effect 1: AS→AP → AIGDD	0.0634	0.0134	0.0393	0.0916	37.7%
Indirect effect 2: AS→AHSB→AIGDD	0.0810	0.0177	0.0491	0.1188	48.2%
Indirect effect 3: AS→AP → AHSB→AIGDD	0.0237	0.0058	0.0137	0.0362	14.1%

SE, standard error; LLCI/ULCI, lower/upper limits of the 95% confidence interval; AS, academic stress; AP, academic procrastination; AHSB, academic help-seeking behavior; AIGDD, AIGD dependency.

## 6 Discussion

This study employed the I-PACE model to investigate the relationship between academic stress and AIGD dependency, with academic procrastination and academic help-seeking behaviors examined as mediators. This study also aimed to understand the motivations behind students’ usage of AIGD, discuss how it is associated with students’ self-perceived competencies, and identify potential risks of AIGD overuse.

Firstly, this research confirms a significant positive association between academic stress and AIGD dependency. Drawing on the stress coping theory (Edwards and Cooper, 1988), individuals are inclined to employ specific coping strategies to alleviate stress-related experiences. This study suggests that for design students, AIGD plays an important role in managing academic stress and task demands. The descriptive findings suggest that some students’ AIGD usage may no longer be limited to academic assistance, but gradually develop a psychological dependence on AI feedback, which may increase their confidence in completing tasks, and improve decision-making. This indicates a shift from instrumental reliance to psychological dependence. This finding aligns with earlier studies conducted by Acosta-Enriquez et al. (2025). The present study extends the research on academic stress and problematic technology use to the emerging context of AIGD (Zhang et al., 2024; Jun and Choi, 2015).

Previous studies have shown that technology dependence in educational contexts mainly includes problematic Internet use, online gaming, social media addiction, and smartphone dependence (Brand et al., 2019; Tang et al., 2014; Wang et al., 2023; Yankouskaya et al., 2025; Zhang and Zhang, 2025). Among these, stress-related mechanisms are particularly significant; academic stress and negative emotions can lead to coping motivation that hinders self-regulation, which is linked to more problematic use behaviors and dependence on technology (Wang et al., 2023; Zhang et al., 2024). This mechanism is crucial for explaining problematic Internet use from a cognitive behavior perspective. Previous research also suggests that avoidance or coping motivations can transform academic stress into problematic smartphone use and online gaming (Sun et al., 2023; Vahedi and Saiphoo, 2018). In this study, the AIGD dependency refers specifically to digital productivity related to learning. During the design learning process, AIGD tools can provide personalized feedback and solutions for students. This use pattern focuses on efficiency and helps explain how academic stress is related to AIGD dependency through procrastination and help-seeking behavior, and explains why AIGD dependency differs from traditional Internet-related problems (Fan et al., 2025).

Accordingly, the study further demonstrated that academic stress is positively associated with academic procrastination, which in turn is linked to higher AIGD dependency. It reveals a dynamic interaction in which stress is accompanied by more negative emotions, which is probably related to greater procrastination as a short-term emotional regulation strategy. This finding is consistent with previous studies (Wahyu Rahardjo et al., 2013). As procrastination accumulates over time, students experience growing pressure to find quick solutions (Li et al., 2020; Xie J. et al., 2023; Yang and Chen, 2025). However, unlike earlier findings of Odaci (2011), the present study found that the AIGD tool’s accessibility and customizability make it efficient for addressing delayed tasks. These features distinguish AIGD from traditional Internet applications, which appear to encourage more pronounced dependency. This finding supports the extension of the I-PACE model’s vulnerability theory to understand technology use in academic settings.

Furthermore, this study demonstrates that high academic stress was associated with AIGD dependency through academic help-seeking behaviors, both direct and indirect pathway which confirms the previous research findings (Shin and Jeon, 2011). Academic help-seeking behavior can help students reduce academic stress and improve well-being. The survey results indicate that AIGD acts as an important medium for academic support; there are differences among students in how they engage with AI-generated results. For instance, whether to modify and optimize the AI-generated content or use it directly. It is important to distinguish between these differences while remaining alert to potential risks to academic integrity. Given the efficiency of AIGD tools, students regard the usage pattern as the preferred path for academic assistance. Without adequate guidance, this reliance may evolve into an entrenched functional dependency. This may be associated with the problem of academic integrity.

Overall, academic stress is related to AIGD dependency by mediating through both academic procrastination and academic help-seeking behavior. The result is consistent with the key perspectives proposed by the cognitive-behavioral framework (Davis, 2001). Academic stress is associated with a greater tendency among students to passive procrastination (Jiang et al., 2021), and the academic difficulties and negative emotions accumulated through procrastination will be linked to greater instrumental help-seeking behaviors, with AIGD serving as a primary form of such behaviors. It functions as an immediate way to alleviate the predicament, eventually fostering technical dependence on AIGD.

Research indicates that the main motivations for design students to use AIGD are centered on functional dimensions, aspects such as improving efficiency, stimulating creativity, and completing academic tasks. This finding is consistent with previous research (Fong et al., 2023; Huang et al., 2024; Jorolan et al., 2025). The study shows that some participants reported high self-perceived competence when AIGD satisfied these functional motivations. The sense of achievement and control brought by overcoming academic barriers with the assistance of AIGD may be the reason for this improvement. This result indicates that the value of AIGD in education extends not only to effectiveness but also beyond functional utility, including the positive shaping of learners’ perceived competencies. This feature highlights the distinction between AIGD and conventional educational tools.

However, this study identified the potential cognitive dependency and skill deterioration risks associated with its use. Some students reported that they experienced mental blocks when they could not use AI tools, which may reflect cognitive dependence during task execution. It indicates that some students may use AIGD to replace essential cognitive processes that ought to be conducted independently, making it difficult to maintain an effective cognitive process without external assistance (Heersmink, 2016). While AI technology offers students a convenient way to obtain information, overuse will bring significant disadvantages (Zhang et al., 2024). Research shows that overreliance on AIGD may be associated with weakened critical thinking and a high risk of academic misconduct (Köbis et al., 2025). Based on this, guiding students toward appropriate AI usage has become an urgent educational concern. This study further discussed AIGD’s different effect, while it functions as an auxiliary tool that is perceived to improve efficiency and is used to cope with stress. However, improper use of AIGD may hinder creativity and deep cognitive development. In addition, AIGD dependency may correlate with social anxiety (Brand et al., 2016). In the process of reliance on AI, some students tend to substitute genuine academic interaction with human-computer communication. This phenomenon deserves further attention in future educational practice.

## 7 Implications

The significant direct and indirect effects observed in the mediation model offer clear theoretical and practical significance.

This study deepens the understanding of technology dependency and problematic behavior within the context of design education. The research sample focuses on design university students; the study provides early investigations into the formation and potential impact of AIGD dependency. The research verified the direct effect of academic stress on AIGD dependency and identified mediating roles of academic procrastination and academic help-seeking behavior. Additionally, the application of the I-PACE model to a generative AI-enabled learning context supports its applicability in explaining AIGD dependency. The findings extend the model’s usefulness to explaining two distinct behaviors of “passive avoidance” (procrastination) and “active response” (help-seeking).

The direct and mediating pathways identified in this study provide actionable strategies for educational intervention. First, educators should pay attention to nurturing students’ critical thinking and independent problem-solving skills, which enhance students’ self-confidence and design abilities to handle complex design tasks. Implementing project-based learning and critical thinking training may help address these demands and potentially reduce the risk of AIGD dependency at its source. Secondly, due to the significant mediating effect of academic procrastination, educators should provide targeted interventions for procrastination and help students establish positive coping mechanisms through time management, goal setting, and other training to avoid relying on AIGD as a compensatory mechanism (Rahardjo et al., 2013). Third, due to the mediating role of academic help-seeking behavior, institutions should establish robust academic support channels, including the enhanced mentor studio system, peer counseling, and psychological support systems (Shin and Jeon, 2011); these resources should encourage students to seek help rather than rely solely on AI technology to deal with academic difficulties. Fourth, educational institutions should conduct targeted AI literacy education programs and ethical guidance to help students use AI generation technology smartly and critically (Chen et al., 2023), to support cautious and appropriate use of AIGD while reducing the risk of dependency. Consistent with the findings, it should be emphasized that AIGD is an auxiliary learning tool, not a substitute for students’ self-thinking and design judgment. Design tasks should be assessed through process-oriented methods such as design iteration, process evaluation, and peer evaluation, rather than just focusing on outcomes. The procrastination-related dependency may be reduced by designing phased deadlines and providing formative feedback on tasks. When educational institutions build an AI-supported academic system, they should attend to students’ cognitive development and social needs while ensuring that this approach cultivate students’ autonomy and learning initiative.

## 8 Limitations and future work

This research has helped reveal AIGD dependency and its underlying mechanisms among design major university students. However, several limitations remain, which provide direction for future research. First, this study adopts a cross-sectional design. Although it clarifies the relationship among variables, it is unable to confirm the causality. Therefore, these findings should be interpreted as evidence of correlation rather than causality. Future research could adopt a longitudinal research design to examine causality and track the effect of AIGD on shaping the core competencies of design students, such as creativity, critical thinking, visual literacy, and creative problem-solving abilities. Such investigations would clarify the profound role of AIGD in design education. Secondly, the results related to perceived competence are based on self-reported rather than objective measures. Therefore, findings about competence improvement and its relationship with AIGD dependency should be treated cautiously. Future studies should use objective measurements to investigate these effects. Third, the study focuses on Chinese design students. Thus, the generalizability of results may be limited by cultural and disciplinary particularities. Future research can adopt cross-cultural and interdisciplinary comparative samples to enhance the generalizability of conclusions. In addition, key variables such as AIGD dependency and academic procrastination were evaluated using self-reported measures, which may lead to potential bias in the research results. Subsequent research should investigate the regulatory factors in various teaching contexts (Freeman et al., 2014). This study treats AIGD dependency as a single concept. The result mainly reflects the tendency toward dependency. It does not distinguish among various usage patterns, such as the rational use of auxiliary tools or the academic misconduct of directly submitting AI-generated content (Xie Y. et al., 2023). Future studies should further investigate these differences to assess the nature and impact of AIGD dependency.

## 9 Conclusion

In summary, this study developed a conceptual model based on the I-PACE framework to examine the formation mechanism of AIGD dependency among design students and empirically analyzed data from 492 Chinese design students. The results show that academic stress has a significant association with AIGD dependency through the chain mediation process of academic procrastination and academic help-seeking behavior. The study further reveals the interaction between academic procrastination and academic help-seeking behavior in the formation of AIGD dependency, providing an empirical basis for understanding the effects of generative AI in educational contexts. Generative AI serves both as a tool for assistance and as a potential source of dependency in educational contexts. In practice, this study offers educators a reference to guide students in cultivating healthy AI usage habits and achieving a balance between improving efficiency and fostering cognitive development. Theoretically, the results provide us with a deeper understanding of student behavior problems in the AI era.

## Data Availability

The raw data supporting the conclusions of this article will be made available by the authors, without undue reservation.

## Footnotes

1
https://www.wjx.cn/
