# Supplementary material for: How academic stress leads to artificial intelligence-generated design dependency: the roles of academic procrastination and help-seeking behavior
Source: Front Psychol. 2026 Mar 18;17:1794730. doi: 10.3389/fpsyg.2026.1794730 (PMC13040471; doi:10.3389/fpsyg.2026.1794730)
Supplement: Supplementary file 1 [file Supplementary_file_1.pdf]

Appendix

Table A1. Variables and items in the questionnaire.

Variables		Items	References
Academic stress	AS1	I am deeply concerned about my academic performance.	Jun and Choi's., 2015
	AS2	Heavy design assignment makes me feel stressed.	
	AS3	My desire to successfully complete my studies makes me feel stressed.	
	AS4	I feel exhausted with my studies.	
Academic procrastination	AP1	I tend to procrastinate when working on design projects	Solomon & Rothblum’s., 1984
	AP2	I often postpone preparation for examination.	
	AP3	I tend to procrastinate when completing course assignments.	
	AP4	I tend to procrastinate when writing academic papers.	
Academic Help-Seeking Behaviour	AHSB1	At least one course I am taking this semester necessitates additional academic support.	Karabenick and Knapp's ., 1988
	AHSB2	In the face of academic challenges, I tend to seek the help of teachers.	
	AHSB3	When I encounter difficulties in my learning, I tend to seek help from peers.	

	AHSB4	When I encounter difficulties in my learning, I tend to seek help from Internet tools.	
AIGD dependency	AIGDD1	I feel uneasy when unable to use AIGD tools.	Morales-García's ., 2024
	AIGDD2	I worry my designs or tasks won't meet expectations without AIGD.	
	AIGDD3	I continuously follow the latest developments in AIGD to ensure that I remain aligned with current trends in the design field.	
	AIGDD4	I feel more confident in my decisions only after receiving feedback from AIGD.	
